# Hyperspectral dark-field microscopy of human breast lumpectomy samples for tumor margin detection in breast-conserving surgery

**DOI:** 10.1117/1.JBO.29.9.093503

**Published:** 2024-05-07

**Authors:** Jeeseong Hwang, Philip Cheney, Stephen C. Kanick, Hanh N. D. Le, David M. McClatchy, Helen Zhang, Nian Liu, Zhan-Qian John Lu, Tae Joon Cho, Kimberly Briggman, David W. Allen, Wendy A. Wells, Brian W. Pogue

**Affiliations:** a National Institute of Standards and Technology, Applied Physics Division, Boulder, Colorado, United States; b Dartmouth College, Thayer School of Engineering, Hanover, New Hampshire, United States; c National Institute of Standards and Technology, Statistical Engineering Division, Gaithersburg, Maryland, United States; d National Institute of Standards and Technology, Materials Measurement Science Division, Gaithersburg, Maryland, United States; e National Institute of Standards and Technology, Sensor Science Division, Gaithersburg, Maryland, United States; f Dartmouth Hitchcock Medical Center, Department of Pathology, Lebanon, New Hampshire, United States; g Battelle Memorial Institute, Columbus, Ohio, United States; h Massachusetts General Hospital, Department of Radiation Oncology, Boston, Massachusetts, United States

**Keywords:** breast tissue imaging, optical medical imaging, hyperspectral imaging, tumor margin detection and imaging, dark field microscopy, spectral unmixing, breast-conserving surgery, image-guided surgery, optical biopsy

## Abstract

**Significance:**

Hyperspectral dark-field microscopy (HSDFM) and data cube analysis algorithms demonstrate successful detection and classification of various tissue types, including carcinoma regions in human post-lumpectomy breast tissues excised during breast-conserving surgeries.

**Aim:**

We expand the application of HSDFM to the classification of tissue types and tumor subtypes in pre-histopathology human breast lumpectomy samples.

**Approach:**

Breast tissues excised during breast-conserving surgeries were imaged by the HSDFM and analyzed. The performance of the HSDFM is evaluated by comparing the backscattering intensity spectra of polystyrene microbead solutions with the Monte Carlo simulation of the experimental data. For classification algorithms, two analysis approaches, a supervised technique based on the spectral angle mapper (SAM) algorithm and an unsupervised technique based on the K-means algorithm are applied to classify various tissue types including carcinoma subtypes. In the supervised technique, the SAM algorithm with manually extracted endmembers guided by H&E annotations is used as reference spectra, allowing for segmentation maps with classified tissue types including carcinoma subtypes.

**Results:**

The manually extracted endmembers of known tissue types and their corresponding threshold spectral correlation angles for classification make a good reference library that validates endmembers computed by the unsupervised K-means algorithm. The unsupervised K-means algorithm, with no *a priori* information, produces abundance maps with dominant endmembers of various tissue types, including carcinoma subtypes of invasive ductal carcinoma and invasive mucinous carcinoma. The two carcinomas’ unique endmembers produced by the two methods agree with each other within <2% residual error margin.

**Conclusions:**

Our report demonstrates a robust procedure for the validation of an unsupervised algorithm with the essential set of parameters based on the ground truth, histopathological information. We have demonstrated that a trained library of the histopathology-guided endmembers and associated threshold spectral correlation angles computed against well-defined reference data cubes serve such parameters. Two classification algorithms, supervised and unsupervised algorithms, are employed to identify regions with carcinoma subtypes of invasive ductal carcinoma and invasive mucinous carcinoma present in the tissues. The two carcinomas’ unique endmembers used by the two methods agree to <2% residual error margin. This library of high quality and collected under an environment with no ambient background may be instrumental to develop or validate more advanced unsupervised data cube analysis algorithms, such as effective neural networks for efficient subtype classification.

## 1 Introduction

Breast-conserving surgery (BCS) is for patients diagnosed with early-stage breast cancer or those who have responded well to neoadjuvant chemotherapy prior to their surgery. While the success rate is high, the risk of BCS is that there could be recurrence, requiring a second surgery with more than 30% rate, for both ipsilateral and systemic cases.^1^ The standard of care requires complete removal of cancerous regions by lumpectomy, leaving no positive margins detected at the time of surgery. The common practice to confirm a successful surgery is a histopathology reading of the hematoxylin and eosin (H&E)-stained slides of the post-lumpectomy sample to confirm a negative or clear margin, where the ductal carcinoma *in situ* should not be found within a 2 mm distance from all specimen edges^2^ or no invasive cancer should be found on any exposed margin surface. With positive or close superficial surgical margins, mastectomy or re-excision should follow within days or weeks, following surgery. The delay in re-excision is sometimes due to the delay in histopathology results. Ideally, an accurate image-guide surgery or an immediate post-lumpectomy evaluation of the margin could be done, to avoid delays to surgery, if positive margins are discovered.

The current gold standard for margin determination is based on histopathological microscopic imaging of the tissue sections stained with H&E. A variety of optical imaging techniques have been demonstrated to enhance breast tumor margin detection in H&E slides of post-lumpectomy samples.^3^ Although the techniques are capable of precise determination of tumor margins, detectable tissue types are limited due to the staining targets nuclei, connective tissue, and fat only. Also, the H&E reading requires the trained eyes of the histopathologists. Label-free imaging methods could avoid the lengthy process involving the H&E staining and reading.

Optical imaging of post-lumpectomy, pre-histopathology samples has been demonstrated by a variety of techniques for imaging a whole sample or partial regions at the suspicious tumor margin. The whole sample imaging techniques include structured illumination imaging, including spatial frequency domain imaging,^4^^–^^7^ near-infrared fluorescence contrast imaging,^8^^–^^11^ photoacoustic tomography,^12^^,^^13^ and terahertz spectroscopic imaging.^14^ Those techniques provide quick assessment of the tumor margin, but they suffer from low spatial resolution and/or insufficient specification in some technologies.^12^ For enhanced spatial resolution, other techniques have been used for imaging partial regions of interest targeting the area at the tumor margin. Such techniques include diffuse reflectance spectroscopy,^15^^–^^17^ spatially resolved Raman spectroscopy,^18^ optical coherence tomography,^19^^,^^20^ light-sheet microscopy,^21^ and nonlinear microscopies (two photon microscopy, second harmonic microscopy, coherent anti-Stokes Raman scattering microscopy).^22^^,^^23^

Hyperspectral imaging (HSI) was initially developed in the field of remote sensing and has been introduced to medical applications to augment conventional spectroscopic imaging technologies. HSI produces images to form a three-dimensional “data cube” consisting of stacked two-dimensional (2D) images of the same scene at multiple contiguous wave bands. More recently, the technologies have increasingly been applied to enable histologic evaluation of tumors in biopsy or post-lumpectomy tissues^7^^,^^24^^–^^27^ to enable intraoperative assessment of tissues. A variety of applications of the HSI for tumor margin imaging have successfully been demonstrated for image-guided clinical treatments involving diabetic wounds,^28^^,^^29^ oral cancer,^30^ head and neck cancer,^25^^,^^27^^,^^31^ and breast cancer.^32^^–^^34^ Extensive discussions on the progress in medical applications of various hyperspectral imaging techniques are available in reviews elsewhere.^35^^–^^38^

Most hyperspectral tumor margin imaging involving HSI technologies and analyses have been demonstrated using reflectance intensity contrast, focusing on classification of the tumor versus all the remaining, non-tumor tissue sites. When classifying multiple tissue types the endmembers, unique spectra of various tissue types, are extracted (supervised) or computed (unsupervised) from a small region of interest in the sample. Then, segmentation maps are established within the same sample from which the endmembers originate. In more sophisticated analysis, multiple thin slices of the same tissue are used for validation by the known number of classes.^39^ However, due to sample-to-sample variations, it is challenging in HSI to build a reliable library of the endmembers of various tumor types that can be used to universally classify tumor regions across other samples. The sample-dependent spectral variation is severe in reflectance mode hyperspectral imaging due to non-negligible sub-pixel mixing with absorption spectra by other non-targeting biological substances (e.g., oxy-hemoglobin). This contribution due to absorption is not uniform across various samples. To mitigate the undesired absorption and multiple scattering spectral contributions, dark-field hyperspectral microscopy been demonstrated for scattering-based imaging of single cells and their subcellular structures,^40^^,^^41^ adipose tissues,^40^ fibrocystic disease, and ductal carcinoma in breast lumpectomy sample^42^ that has reported discernible contrast in scattering power between the benign versus malignant tissues.

The primary objective in analyzing a data cube is to ascertain the presence or absence of a specific target tissue type with a known spectral signature within the data cube. Various classification algorithms, both supervised and unsupervised, have been showcased for this purpose. Extensive discussions on the details and pros and cons of various algorithms are reviewed elsewhere.^43^^–^^46^ The algorithms are divided into two categories: supervised and unsupervised. The supervised approach or signature-based target detection relies on *a priori* spectral information of specific tissue types as reference endmembers, so-called “expert labels,” and employs classification algorithms to identify a tumor region with a spectrum similar to its reference or expert label. Such algorithms include support vector machine with feature-selection technique for tumor detection^39^^,^^47^^,^^48^ supervised spectral-spatial Fisher’s linear discrimination analysis,^49^ spectral slope classification method,^50^ artificial neural networks,^51^ and spectral angle mapper (SAM).^52^ On the other hand, the unsupervised approach is based on a purely statistical computation with no *a priori* spectral information of the target tissue types, and such algorithms include principal component analysis,^53^ non-negative matrix factorization,^54^ hierarchical-distributed stochastic neighbor embedding algorithm,^55^ and K-means clustering.^56^ Among other unsupervised approaches, the K-means clustering is one of the most intuitive algorithms, providing clusters of pixels based on their spectral similarity under an assumption that the spectral signatures of pixels belonging to the same cluster are assumed to approximately lie in a low-dimensional subspace.^44^ Furthermore, based on endmembers returned by the K-means, solving non-negative least squares problems quantifies each pixel’s spectral similarity to the computed endmember of each cluster to which the pixel belongs, allowing for spectral unmixing when multiple endmembers are associated with that pixel.

This work expands the application of hyperspectral dark-field microscopy (HSDFM) to the classification of tissue types and tumor subtypes in pre-histopathology human breast lumpectomy samples. Two analysis approaches, a supervised technique based on the SAM algorithm and an unsupervised technique based on the K-means algorithm, are applied to classify various tissue types including carcinoma sub types. In the supervised technique, the SAM algorithm with manually extracted endmembers guided by H&E annotations is used as reference spectra, allowing for segmentation maps with classified tissue types including carcinoma subtypes. The manually extracted endmembers of known tissue types and their corresponding threshold spectral correlation angles (SCA) for classification make a good reference library that validates endmembers computed by the unsupervised K-means algorithm. The unsupervised K-means algorithm with no *a priori* information produces abundance maps with dominant endmembers of various tissue types, including carcinoma subtypes of invasive ductal carcinoma (IDC) and invasive mucinous carcinoma (IMC).

## 2 Methods

### 2.1 Hyperspectral Dark-Field Microscopy

**Fig. 1:**
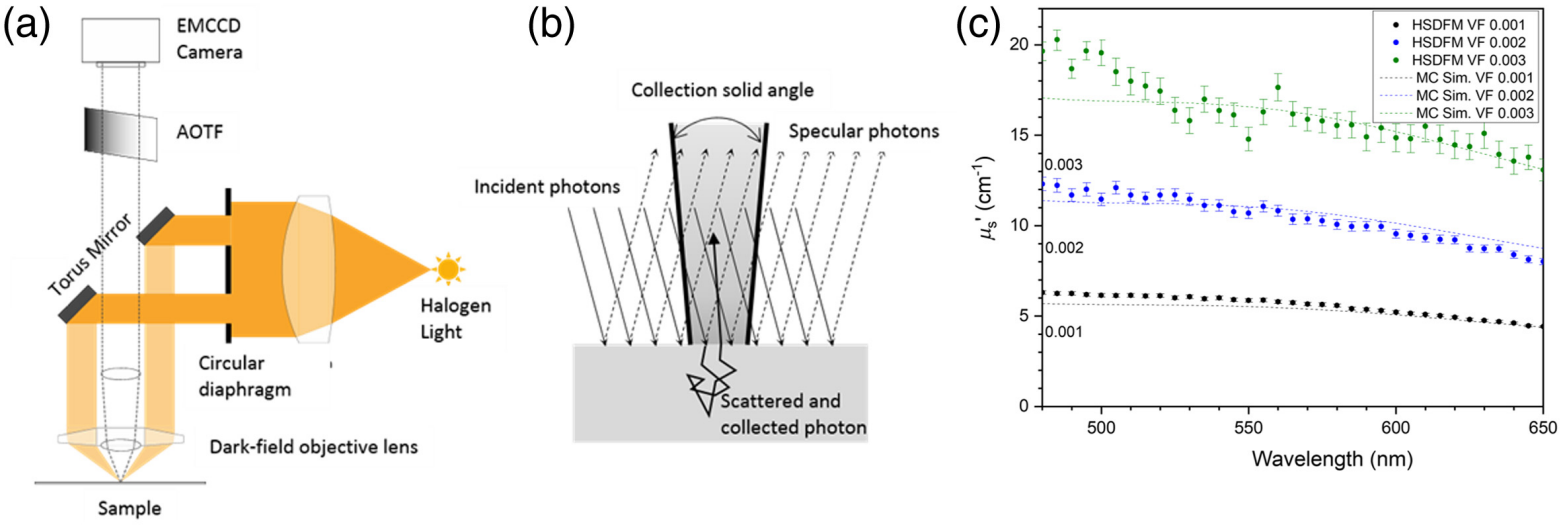
Schematic of the hyperspectral dark field microscope and its performance validation. (a) Schematic of HSDFM setup. (b) Configuration for the Monte Carlo simulation, (c) reduced scattering coefficient spectra of three polystyrene solutions at different volume fractions. The dotted lines are simulated look-up table results by the Monte Carlo simulation at various volume fractions of the model microbead solutions. The colored data points are reduced scattering coefficient spectra of the three different bead solutions, calculated by the Monte Carlo simulation using the Mie phase function defined by the size and concentration of each bead solution.

The details of our HSDFM setup are described elsewhere^41^ and its schematic is shown in Fig. 1(a). The HSDM is an optical microscope (Olympus BX60) with a broadband white halogen lamp (100W HAL-L 7724 bulb, Phillips). The lamp is collimated through a condenser lens and illuminates the sample through a ring mask to form a conically soft focused beam through a 5× objective lens with numerical aperture of 0.13 (UIS 2 LMPlanFL N, 5X, NA = 0.13, Olympus).

The essence of the HSDFM is an incident illumination angle larger than the maximum collection angle of the objective lens, which is $\sin^{-1}(NA/n)$, where NA is the numerical aperture of the imaging objective lens and n is refractive index of the medium in the beam path. This configuration allows for an effective rejection of specular reflectance by the imaging objective lens. The image contrast comes primarily from the back-scattered light in the upper layers of the sample. Reflected light collected by the objective passes through an opto-acoustic tunable filter (AOTF, HSI 300, Gooch & Housego) and is focused to an electron-multiplying charge-coupled device (EMCCD) camera (iXon 897, Andor). A calibration procedure to account for the illumination spectrum of the lamp, the spectral transmission of the AOTF and optical path, and the spectral response of the EMCCD is described elsewhere.^57^ HSDFM data cubes are a set of snapshot images acquired at each wavelength from 480 to 650 nm at every 5 nm wavelength step with 8 nm bandwidth. To acquire multispectral images without camera saturation, the exposure time of the EMCCD was controlled by codes to maximize the dynamic range (about 95% fill factor) of the camera at a fixed gain during the acquisition of the entire data cube. The intensity histogram at every wavelength was examined at various exposure times before data storage to select the optimum exposure time which results in the maximum dynamic range. This procedure avoids gain-dependent nonlinear responsivity of the camera sensor. The range of the exposure time to scan was pre-determined by the lamp’s spectral intensity and the camera’s spectral responsivity to avoid a lengthy exposure adjustment process, to allow for acquisitions in a few seconds at each wavelength up to 650 nm. We found the exposure adjustment time above 650 nm and NIR range takes longer than a minute as the silicon EMCCD camera’s quantum yield is relatively low in this range.

The performance of the HSDFM was evaluated by analyzing back scattering intensity spectra from liquid phantoms of water dispersed with polystyrene spheres of 368 nm diameter (Polybead Cat # 07306). Three samples at nominal volume fractions (volume of spheres/volume of water) of 0.003, 0.002, and 0.001 (polystyrene/water) were prepared by dilution and were imaged. For spectral measurements of the microsphere samples, the scattering intensity was averaged over a 100 pixel by 100 pixel region of interest at the center of the field of view (FOV) at each wavelength. The reflectance spectra of the three liquid phantoms were compared with a series of spectra (back reflected intensity versus wavelength) generated by a Monte Carlo simulation at various volume fractions of the PS beads in water. Monte Carlo simulations of photon transport for the HSDFM were done by a customized computed unified device architecture Monte Carlo maximum likelihood code^58^ running on a graphical processor unit (Nvidia Tesla M2050 and GeForce GTX960). In the simulation implementing the dark field microscope configuration, photons from a planar source were injected at a 15 deg incident angle onto the sample surface of 5 mm diameter, and the back scattered photons were collected with the exit angles smaller than $\sin^{-1}(\mathrm{NA}/n)$, where NA is the numerical aperture of the objective lens, from a 1.6 mm diameter FOV area. A schematic of the simulation geometry is shown in Fig. 1(b). Each simulation was performed with 100 million incident photons and with the upper limit of one million collected photons. From the simulation, the reflectance value, the ratio of the number of collected photons versus the injected photons, was recorded. From the nominal microsphere concentration, the volume fractions for a series diluted samples were calculated. A look-up table (LUT) of simulated reflectance curves was established with volume fractions ranging from 0.0005 to 0.005 in steps of 0.0005 and the absorption coefficients set to zero according to the following procedure. The z-average size (diameter) of polystyrene spheres in a 0.3% solution by volume was measured by a dynamic light scattering system (Zetasizer Nano, Malvern)^59^^,^^60^ and was found to be 368.0±3.7  nm (one standard deviation by five measurements) with monomodal size distribution (polydispersity <0.1). The Mie scattering phase function for simulation was calculated for 368 nm diameter polystyrene spheres at different wavelengths (from 400 to 800 nm in 25 nm steps) as well as with different volume fractions. The absorption coefficients were set to zero and the wavelength-dependent anisotropy, g(λ), and reduced scattering coefficient, $\mu_{s}^{\prime }(\lambda )=\mu_{s}(\lambda )(1-g(\lambda ))$, were calculated using an algorithm published elsewhere^61^^,^^62^ and were used for simulations to establish an LUT of reflectance versus wavelength curves for different volume fractions. An LUT of $\mu_{s}^{\prime }(\lambda )$ at various volume fractions were also established from the calculation for the inverse solution of the $\mu_{s}^{\prime }(\lambda )$ of the liquid phantoms as shown in Fig. 1(c). The three $\mu_{s}^{\prime }(\lambda )$ of different volume fraction samples obtained from the HSDFM agree well with the LUT $\mu_{s}^{\prime }(\lambda )$ and verifies that the intensity of the $\mu_{s}^{\prime }(\lambda )$ at each wavelength is proportional to the volume fraction (i.e., relative concentration) of the PS beads.

An acquired HSDM image is a reflectance intensity data cube, I(m,n,λ). The estimated spatial resolution of 10.2±2.1  μm of the HSDM in a broadband was determined by analyzing a data cube of a USAF 1951 resolution target (DA007, Edmund Optics), as discussed in detail in Fig. S3 in the Supplemental Material. For each sample, a data cube of a reflectance standard (Spectralon Labsphere) was acquired under the same conditions, and its averaged spectrum, $I_{\mathrm{ref}}(\lambda )$, across the FOV was used for normalizing the multispectral data cube. An initial data processing involving normalization and correction with the dark background spectrum, $I_{\text{dark}}(\lambda )$, builds a data cube $\vec{X}(m,n)$ to analyze:

$$\overset{\to }{X}(m,n)=X(m,n,\lambda )=\frac{I(m,n,\lambda )/\Delta \tau_{1}(\lambda )-I_{\text{dark}}(\lambda )/\Delta \tau_{2}(\lambda )}{I_{\mathrm{ref}}(\lambda )/\Delta \tau_{3}(\lambda )-I_{\text{dark}}(\lambda )/\Delta \tau_{4}(\lambda )},$$ (1)

where each intensity map was normalized by its exposure time, $\Delta \tau_{n}(\lambda )$ at every wavelength for cube n (note that $\Delta \tau_{n}(\lambda )$ for the data cubes in Eq. (1) are not the same), and the data cube $\vec{X}(m,n)$ was processed further for spatial flattening to correct any long-range non-uniform illumination across the FOV due to sample tilt and spatial nonuniformity at certain wavelengths. The Gaussian smoothing algorithm with the directional intensity standard deviation across the image was used for this correction in an image analysis package. For tissue image collection, a resected tissue sample was placed onto a sterile tissue-mounting cartridge and covered with a glass slide to mitigate potential surface roughness-dependent artifacts. To retain moisture in the tissue sample, the total time to handle the sample and to acquire the data cubes for all FOVs was limited to a few minutes for each sample. During the data collection, the whole microscope was contained within a light-shield curtain to reject any ambient light.

#### 2.2 Analysis Algorithm Codes

In hyperspectral data analysis, the spectrum of a pixel (m,n) in the hyperspectral data cube is treated as a linear superposition of multiple spectra of “pure” individual substances called “endmembers.” In HSDFM, the endmembers are the back scattering intensity spectra from cellular and molecular substances within each pixel. For the supervised approach, the endmembers in this study were extracted from selected regions of known tissue types,^63^ where the tissue type information was provided by the histopathology annotation for specific regions. Computation of the SCA and the segmentation map associated with each extracted endmembers were done by the SAM code by a commercial software package, ENVI (version 6.0, Harris Geospatial Solutions). Details of the endmember extraction and SCA computation procedures are described in the results and discussion section. Details of the K-means algorithm are described in Sec. 3 as well. Both supervised and unsupervised analyses were performed in the Microsoft Windows 10 operating system (Microsoft Corp., Redmond, Washington).

#### 2.3 Tissue Sample Preparation and Histopathology Imaging

Freshly resected human breast tissue was imaged at the Dartmouth-Hitchcock Medical Center under an IRB-approved protocol. The fresh tissue sample was placed on a sterile petri dish and covered by a glass slide to mitigate artifactual signal due to the surface roughness of the tissue. Each sample was photographed using a digital camera installed with a light illumination station, followed by HSDFM imaging. After imaging, the sample was contained in saline solution at 4°C until H&E slide preparation for digital imaging and for digitally marking with annotations on the image. H&E slides were prepared by a procedure described in detail elsewhere.^4^ In brief, tissues were fixed in 10% buffered formalin (Biochemical Science Inc., Swedesboro, New Jersey) and dehydrated through graded alcohols, followed by paraffin embedding. Then, the embedded tissue was sectioned into slices of 4  μm thickness and mounted onto a glass slide with adhesive (Sta-on; Surgipath Medical Industries, Richmond, Illinois). The mounted slide was stained with H&E followed by air-drying for at least 30 min and loading onto a Leica Bond Max automated immunostainer (Leica Biosystems, Deer Park, Illinois). Then the mounted sample was baked (30 min at 60°C) and dewaxed for 30 min at 72°C, while being rinsed with alcohol, then washed in bond wash buffer (DMI Medical Inc., Davie, Florida).

## 3 Results and Discussion

### 3.1 Extraction of Endmembers of Several Types from the Data Cubes Acquired by the Hyperspectral Dark-Field Microscope

**Fig. 2:**
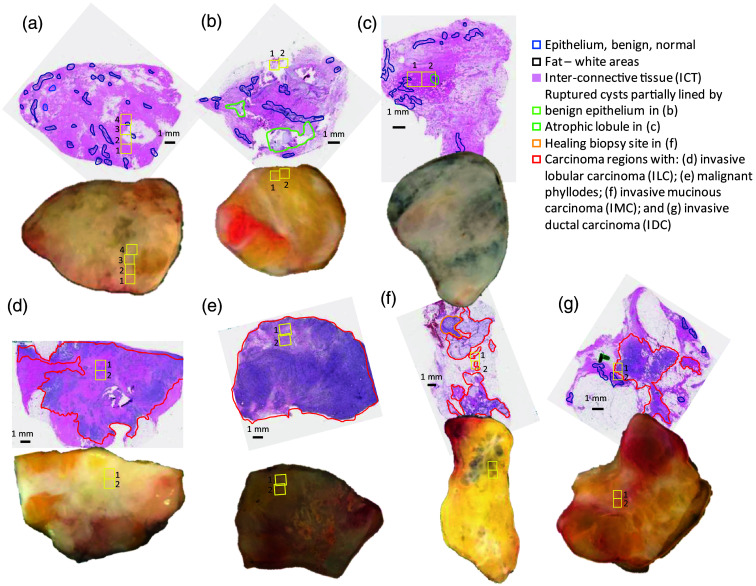
Lumpectomy samples of various tissue types. Each panel in Fig. 2 compares a digital photograph (bottom) and its companion H&E slide micrograph (top) for each sample to identify as: (a) normal with benign epithelium; (b) normal with benign epithelium and ruptured cysts; (c) normal with benign epithelium with atrophic lobules; (d) abnormal with invasive lobular carcinoma; (e) abnormal with malignant phyllodes; (f) abnormal invasive mucinous carcinoma; (g) abnormal with invasive ductal carcinoma. The carcinoma regions are annotated by red enclosure lines on the H&E slide images. Inside the yellow boxes are FOVs from which data cube data were acquired. On the H&E slides, the green enclosed regions in (b) and (c) are ruptured cysts and atrophic lobules, respectively, and the orange enclosed region in (f) a healing biopsy site.

**Fig. 3:**
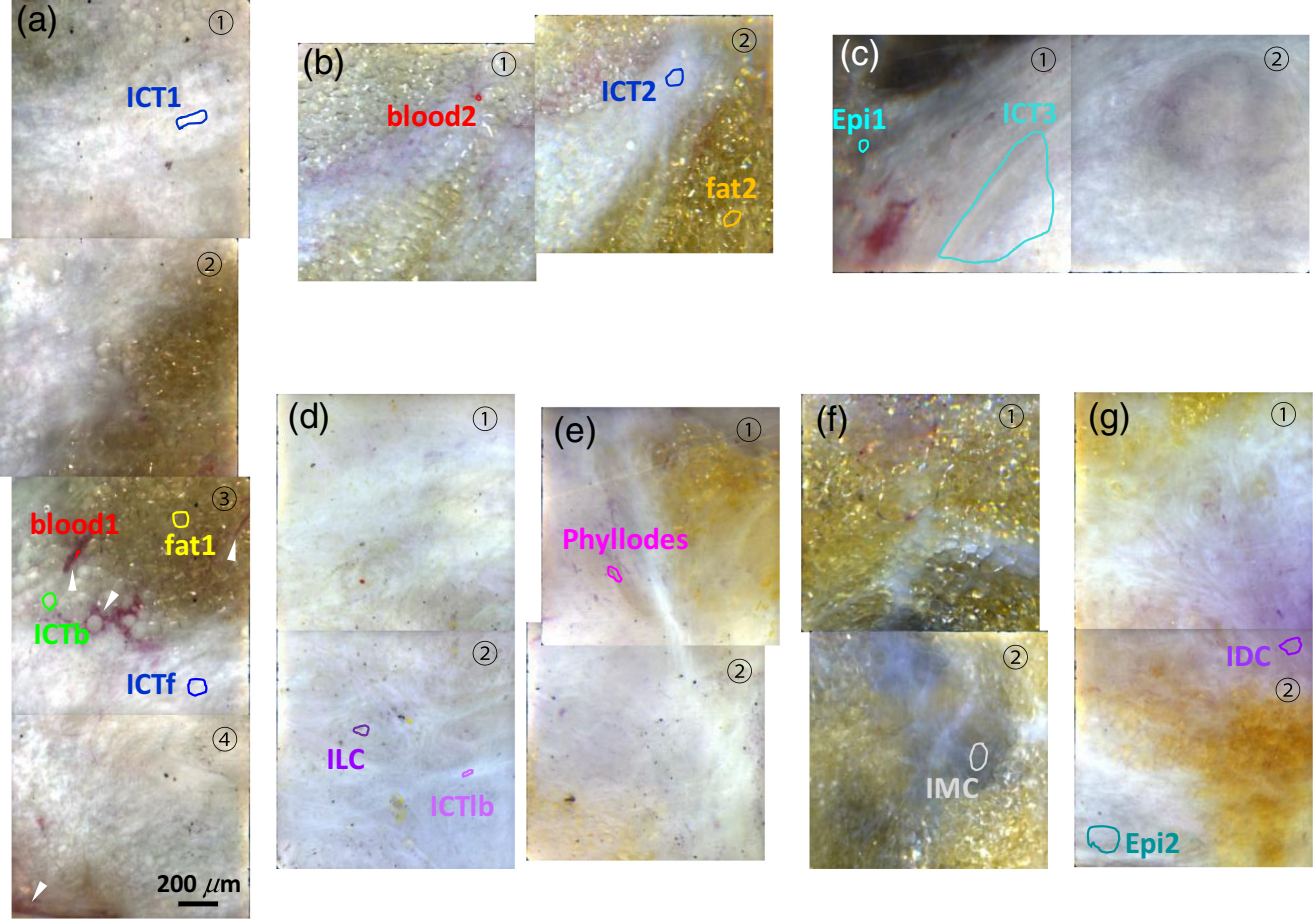
Pseudo color images of the HSDFM data cubes. A collection of contiguous pseudo color images processed from HSDFM data cubes of the FOVs marked on the images shown in Fig. 2. Regions from which the endmembers for various tissue types are marked on the images. The same color for each tissue type is used for the label in this figure, endmember plots, and segmentation maps throughout this paper. The circled numbers indicate the FOV numbers marked in Fig. 2.

The HSDFM images of human breast lumpectomy samples were excised from multiple patients during BCS. Each panel in Fig. 2 compares a digital photograph (bottom) and its companion H&E slide micrograph (top) for each sample. The H&E slide preparation and its imaging were done after acquiring a digital photograph immediately followed by the HSDFM acquisition to ensure the spectral characteristics of the fresh wet tissue are included in the data cube. The FOV of an HSDFM data cube is limited to a 1.2  mm×1.2  mm, about 100 times smaller than the total area of the tissue, therefore the data acquisition locations of the FOVs were determined by the local color variation to include as many tissue types as possible within the normal samples in Figs. 2(a)–2(d) or to include the suspected tumor boundary with the tumor-include samples in Figs. 2(d)–2(g). Two or four data cubes were collected from contiguous FOVs to analyze contiguous features across the selected FOVs, and their locations are marked with yellow squares both on the digital photographs and on the H&E slide images. The continuity test of those contiguous features in the analyzed classification maps is one of the test methods to check the validity of the extracted endmembers which is discussed later in depth. The H&E slide preparation involves a series of procedures including chemical fixation, paraffin embedment, and slicing, causing target features’ microscopic distortions and displacement from the initial locations in the pre-treated sample. Therefore, the locations of the corresponding FOVs on the companion H&E image were determined by visually matching the corresponding features between the pseudo-color images (PCIs) in Fig. 3 and their companion H&E slide images in Fig. 2. For example, the patterns of interconnected tissue (ICT, pink) and adipose or fat tissue (white) in the H&E slide were identified to place the data cube’s FOV locations on the H&E slides by matching the patterns of gray (for ICT) and yellow color (for fat) in the corresponding PCI in Fig. 3(a). To construct the PCI, three images at 480, 530, and 630 nm bands were selected from each data cube for the blue, green, and red channels, respectively. Then each channel’s intensity value was assigned to each element in the vector (b, g, r) to build a pseudo color for each pixel.

The H&E staining identifies regions of carcinoma subtypes as well as specific tissue types in the lumpectomy tissues. The tissue samples shown in Figs. 2 and 3 are (a) normal with benign epithelium, (b) normal with benign epithelium and ruptured cysts, (c) normal with benign epithelium with atrophic lobules, (d) abnormal with invasive lobular carcinoma (ILC), (e) abnormal with malignant phyllodes, (f) abnormal with IMC, (g) abnormal with IDC. The carcinoma regions are encircled by red lines on the H&E slide images. The yellow boxes are FOVs from which data cube data were acquired. The H&E micrographs provide rich anatomical details with common breast tissue features identified both in normal and tumor-containing samples. Such features include spatial distributions of ICT, fat, and epithelium identified as pink and white, respectively, and their regions enclosed by blue lines, respectively, by a histopathologist. Furthermore, the H&E micrographs also revealed other prognosis features, such as ruptured cysts partially lined by benign epithelium (green in (b)), highly atrophic lobule (green in (c)), and a healing post-biopsy site (orange in (f)).

The H&E staining identifies the spatial distribution of each tumor subtype. Lobular carcinoma *in situ* is a precancerous condition characterized by the presence of abnormal cells within the milk glands, posing a risk of progressing into malignant cancer outside of the gland.^64^ When the cancer cells break out of the lobule, they invade into neighboring tissue, resulting in spreading tumor cells associating with ICT as shown in (d) with an ILC region with the size of a few centimeters. The PCIs in Fig. 3(d) display interspersed dark spots indicative of tumor cells widely spread across the ICT. Malignant phyllodes tumor in (e) is a fibroepithelial neoplasm developed in the ICT, not from ducts or glands from which most breast tumors originate. The corresponding H&E slide shows cellular spindle-cell neoplasm with benign residual epithelial components.^65^ The PCIs in Fig. 3(e) identify dark tumor cells associated with the broad ICT region. IMC in Fig. 3(f) is another relatively rare carcinoma subtype and is characterized by a large amount of extracellular mucin which usually is shown as a dark hazy pattern on the tissue under a white light illumination, indicative of hypocellular mucinous carcinoma.^66^ Lastly, IDC in Fig. 3(g) is the most common, about 75% to 80% of all breast cancers.^67^ This carcinoma arises from the milk duct then radiates into the surrounding breast tissue rich in ICT and fat, usually forming an infiltrating solid mass. PCIs in Fig. 3(g) show a pinkish region colocalized with spindle cell nuclei associated with gray ICT regions, characterized by a radiating fibrous pattern running diagonally from the right middle.

**Fig. 4:**
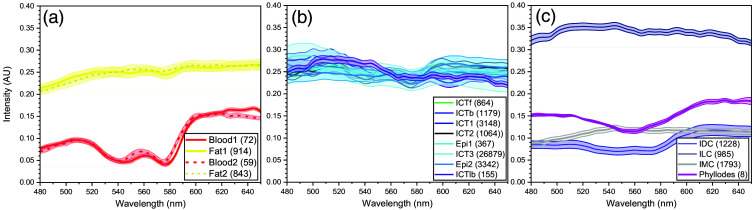
A collection of endmembers of various tissue types. A collection of endmembers of various tissue types extracted from the regions marked on the pseudo color images of different tissue samples shown in Fig. 3: (a) fat and blood endmembers; (b) ICTs, and epithelia; and (c) carcinoma subtypes. The standard deviation of each spectrum is defined by the vertical height of the shaded region of each spectrum. The numbers in parentheses in the legends are the number of pixels from which each endmember was extracted.

**Fig. 5:**
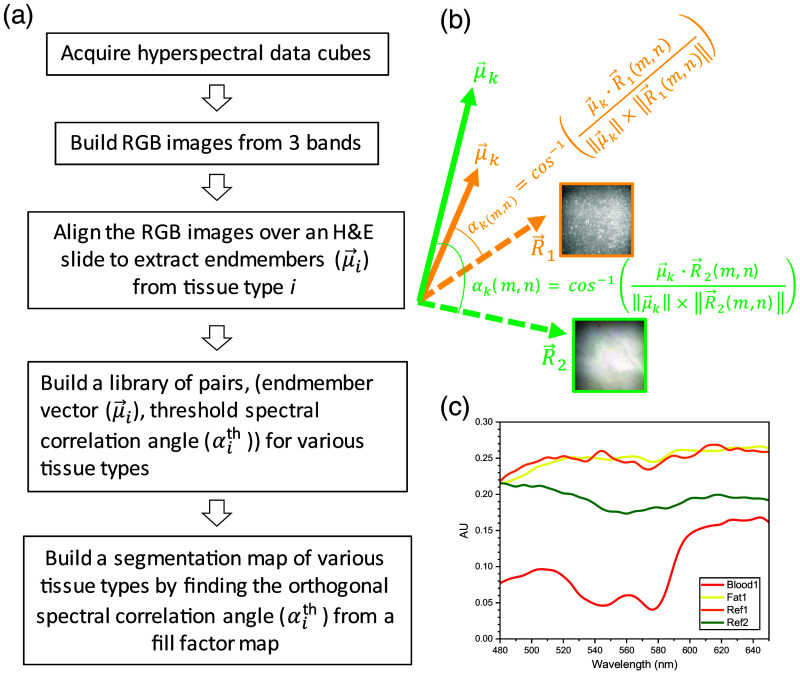
Procedure to determine the orthogonalized spectral correlation angle, OSCA. (a) The flowchart of the procedure. (b) Two reference data cubes used to determine the OSCA for the endmember of each tissue type. (c) The mean spectra of the reference data cubes and endmembers of “blood1” and “fat1” are displayed.

Figure 3 shows a collection of contiguous PCIs from the data cubes of the FOVs marked in Fig. 2. The annotated tissue patterns within the corresponding FOVs on the companion H&E slide allow for visual classification of tissue types in the PCIs. For example, the patterns with gray and yellow regions in the PCIs in a normal tissue shown in Fig. 3(a) match to ICT and fat regions in the companion H&E slide image in Fig. 2(a), respectively. The red tubular and elongated features (white arrowhead) are blood vessels which are not identified in the corresponding H&E slides. On the PCIs, the regions from which spectral endmembers shown in Fig. 5 for different tissue types and blood vessels are marked with various colors for their boundaries: only one region is selected for blood and fat, but several regions for the ICT are selected as the ICT distribution is relatively broader. ICTb, ICTf, and ICT are from regions close to the blood vessel, near the fat tissue, and far from both, respectively. To extract additional endmembers from other tissue samples, PCIs and their companion H&E slide images were compared to label a tissue type to each extracted endmember: blood, fat, and ICT endmembers from the normal tissue in Fig. 2(b) are labeled with blood2, fat2, and ICT2, respectively. Likewise, the comparison between colored features in the PCIs and their companion H&E slide images allowed for locating regions with carcinoma subtypes from which endmembers for the subtypes were extracted and labeled. The same color for each tissue type is used for the ROI boundary in Fig. 3, endmember plots in Fig. 4, and supervised segmentation maps in Figs. 7–9. Note that the colors assigned for tumor subtypes in those figures are different.

Figure 4 shows a collection of endmembers and associated standard deviations of various tissue types extracted from the regions marked on the PCIs in Fig. 3. Each endmember, ${\vec{\mu }}_{i}$ in this plot is a mean of multiple spectra from pixels within each marked region whose number of pixels is shown in the parenthesis of each tissue types in the figure legends. Two endmembers for blood and fat tissues from different normal tissue samples shown in Figs. 2(a) and 2(b), elucidating that the two spectra from different samples is similar to each other. The fat spectra are nearly flat above 550 nm but with scattering intensity decreasing as the wavelength decreases below 550 nm. The blood spectra share the same feature with local minima at about 540 and 575 nm which correspond to two absorption peaks of oxygenated Hb of blood. This result implies that the HSDFM acquisition on fresh tissues is still susceptible to light absorption, implying the collected backscattering signal in the blood region may include multi-scattering signal which involves long scattering pathlength. Two blood spectra shown are slightly different both in the local minima and in the slope above 600 nm, indicative of tissue-to-tissue variations in scattering signal and partial oxygen pressure convoluted together. The spatial distribution of the ICT appears so broad that we extracted ICT endmembers from various regions of one sample [Fig. 3(a)] and from another normal sample, Fig. 3(b), as well as from a tumor-including sample, Fig. 3(d). For example, ICTf and ICTb are from regions in the proximity of fat and blood, respectively, in the benign fibroid sample in Fig. 3(a) and ICT1 from a region far away from both. Figure 4(b) shows a collection of those ICT endmembers extracted from those ROIs. They all share the similar pattern with relatively small intensity fluctuations with an overall negative slope versus wavelength, implying that ICTs are characterized with relatively uniform scattering intensities across wavelengths, quite distinct from those of fat and blood. The 3% to 4% fluctuation in the ICT spectra are not prominent in other samples, indicative of backscattering intensity modulation unique in the ICT regions. In the same plot with ICT spectra, two epithelium spectra (cyan), “epi1” from a normal tissue and “epi2” from IDC tissue are shown for comparison, indicative of similar spectra to those of ICTs but with less local fluctuations. Further measurements with connective tissue samples would need to clarify the exact source of this fluctuation and to quantify the signal. Figure 4(c) compares four spectra extracted from regions identified with different carcinoma subtypes, ILC, phyllodes, IMC, and IDC. The spectra of IDC (purple) and phyllodes (pink) appear to be similar with decreased intensities in 510 to 610 nm, but the IDC spectrum shows less curvature in that wavelength region. On the other hand, ILC and IMC spectra have a concave down shape with local maxima at different wavelengths. These distinct spectral features of various carcinomas allow for segmentation maps of different subtypes. Details on building segmentation carcinoma maps using these spectra are discussed in the next section.

### 3.2 Supervised Algorithm to Build Segmentation Maps Delineating Different Tissue Types and Carcinoma Subtypes, Based on Endmembers’ Orthogonalized Spectral Correlation Angles

Our analysis goal is to obtain spatial segmentation maps of various tissue types from a 3D intensity data cube I(m,n,λ) or a data cube $\vec{X}(m,n)$ and to ultimately classify tumor regions and their boundaries. Discriminating pixels with a specific tissue’s endmember is challenging in a data cube of biological tissues due to complex sub-pixel mixing of multiple endmembers. Our supervised approach here focuses on the classification of a specific tissue type by determining which extracted endmember is of the closest match to the spectrum at each pixel.

To this end, our supervised approach is based on the SAM algorithm which has been introduced to compute the degree of similarity between $\vec{X}(m,n)$, a spectrum at pixel (m,n), and ${\vec{\mu }}_{i}$, the endmember of a specific element (tissue) type i.^68^ For tissue type classification, the SAM treats the spectra as vectors in a hyperspectral space with dimensionality equal to the number of wavelength bands.^35^ As illustrated for the SAM algorithm in Fig. 5(b), the spectral vectors can be placed along the lines that pass through the origin of the multi-dimensional hyperspace. For a tissue type i, the SAM calculates $\alpha_{i}(m,n)$, or the SCA (in radian) between $\vec{X}(m,n)$ and ${\vec{\mu }}_{i}$:

$$\alpha_{i}(m,n)=\cos^{-1}(\frac{\overset{\to }{X}(m,n)\cdot {\overset{\to }{\mu }}_{i}}{\left\|\overset{\to }{X}(m,n)\right\|\times \|{\overset{\to }{\mu }}_{i}\|}).$$ (2)

Note that calculation of SCA involves normalization with the vectors’ amplitudes, making it insensitive to the uniform rescaling across the wavelength. Therefore, $\vec{X}(m,n)$ and ${\vec{\mu }}_{i}$ can be rescaled by a constant multiplication factor without changing the result. The SCA, between 0 and π/2, quantifies the similarity between $\vec{X}(m,n)$ and ${\vec{\mu }}_{i}$: the smaller the SMA value, the closer the match.^69^

With a given threshold SCA (TSCA), $\alpha_{i}^{\mathrm{th}}$, we define a segmentation map for a tissue type i as

$$I_{i}^{\mathrm{seg}}(m,n,\alpha_{1,i})=\{\begin{array}{cc} 0; & \alpha_{i}(m,n)\geq \alpha_{i}^{\mathrm{th}}\\ 1; & \alpha_{i}(m,n)<\alpha_{i}^{\mathrm{th}} \end{array}.$$ (3)

Ideally, the $\alpha_{i}^{\mathrm{th}}$ is determined by the relation: $\|I_{i}^{\mathrm{seg}}(m,n)-I_{i}^{\mathrm{gt}}(m,n)\|=\text{null}$, where $I_{i}^{\mathrm{gt}}(m,n)$ is a corresponding ground-truth image in which the spatial information of the tissue type is completely known. One would think that $I_{i}^{\mathrm{gt}}(m,n)$ can be obtained from the counterpart H&E slide’s image, but it is not readily usable for this computation because pixel-to-pixel matching between an H&E image and its companion data cube is not possible: preparation procedure of H&E slides, involving chemical fixation and slicing, result in microscopic morphological changes. Alternatively, we define $\alpha_{i}^{\mathrm{ort}}$, the orthogonalized SCA (OSCA) with which we build segmentation maps instead with $\alpha_{i}^{\mathrm{th}}$. The procedure to determine the OSCA is summarized in a flowchart in Fig. 5(a), and the details are explained below.

In essence, $\alpha_{i}^{\mathrm{ort}}$ for an endmember (${\overset{\to }{\mu }}_{i}$) is determined against its reference data cube to make ${\vec{\mu }}_{i}$ orthogonal to it. The reference data cube is ${\vec{R}}_{l}(m,n)$, where l=1,2 so that two 2D reference spectral vectors, ${\overset{\to }{R}}_{1}(m,n)$ and ${\overset{\to }{R}}_{2}(m,n)$ are data cubes of fat only and of fibroadenoma only, respectively, where this information is confirmed by H&E reading. ${\overset{\to }{R}}_{1}(m,n)$ was used to determine $\alpha_{i}^{\mathrm{ort}}$ for endmembers of non-fat tissues (${\overset{\to }{\mu }}_{i};i\neq \text{fat}$) and ${\vec{R}}_{2}(m,n)$ for fat tissue endmembers, (${\overset{\to }{\mu }}_{i};i=\text{fat}$). The PCIs of these two reference data cubes are displayed in Fig. 5(b) and their mean spectra across the FOV in Fig. 5(c) along with ${\overset{\to }{\mu }}_{\text{blood}1}$ and ${\overset{\to }{\mu }}_{\text{fat}1}$ for comparison.

The $\alpha_{i}^{\mathrm{ort}}$ is determined from a fill factor map which is calculated by the SMA algorithm against the reference data cube so that

$$F_{i}(m,n)=\{\begin{array}{cc} 1; & \beta_{i}(m,n)\geq \alpha_{i}^{\mathrm{ort}}\\ 0; & \beta_{i}(m,n)<\alpha_{i}^{\mathrm{ort}} \end{array},$$ (4)

where

$$\beta_{i}(m,n)=\cos^{-1}(\frac{{\overset{\to }{\mu }}_{i}\cdot {\overset{\to }{R}}_{l}(m,n)}{\left\|{\overset{\to }{\mu }}_{i}\right\|\times \|{\overset{\to }{R}}_{l}(m,n)\|}),$$ (5)

and l=1 for k≠fat, and l=2 for k=fat. From this fill factor map, the fill factor, the ratio, number of non-zero pixels against the total pixel numbers is defined by the following histogram:

$$f_{i}(\overset{¯}{\beta_{i}})=\frac{1}{N}\underset{m,n}{\sum }F_{i}(m,n),$$ (6)

where N is the total number of pixels in a data cube, and $\overset{¯}{\beta_{i}}$ in Eq. (6) is a scalar variable defined in Eq. (4).

**Fig. 6:**
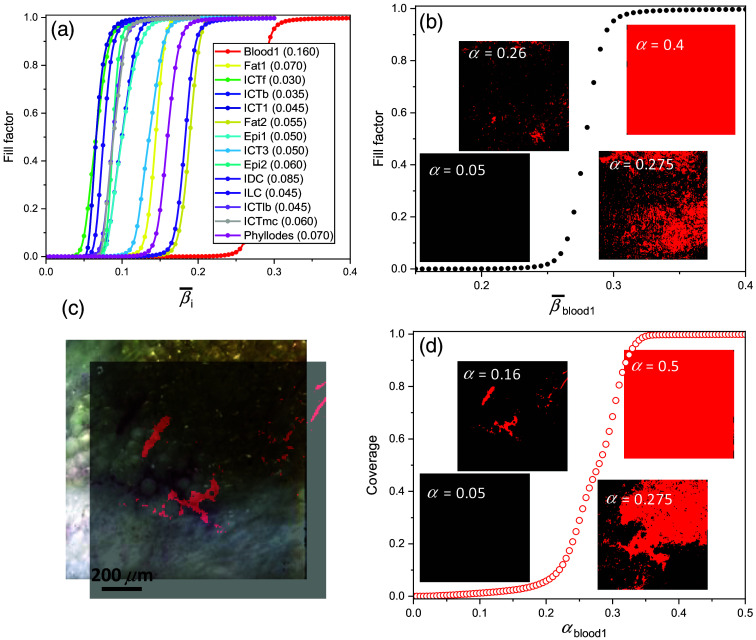
The fill factor versus SCA and blood segmentation maps. (a) The fill factor versus spectral correlation angle of various tissue types. (b) A plot of $f_{\text{blood}1}({\overset{¯}{\beta }}_{\text{blood}1})$) along with $F_{\text{blood}1}$ (m,n) map at several ${\overset{¯}{\beta }}_{\text{blood}1}$ values. (c) A segmentation map of “blood1” is overlaid onto the corresponding PCI in Fig. 3(a) FOV3. The calculated blood segmentation map is diagonally translated 50×50  pixels for a better visibility. (d) Calculated coverage versus $\alpha_{\mathrm{blood}1}$ and several segmentation maps at various $\alpha_{\text{blood}1}$.

**Fig. 7:**
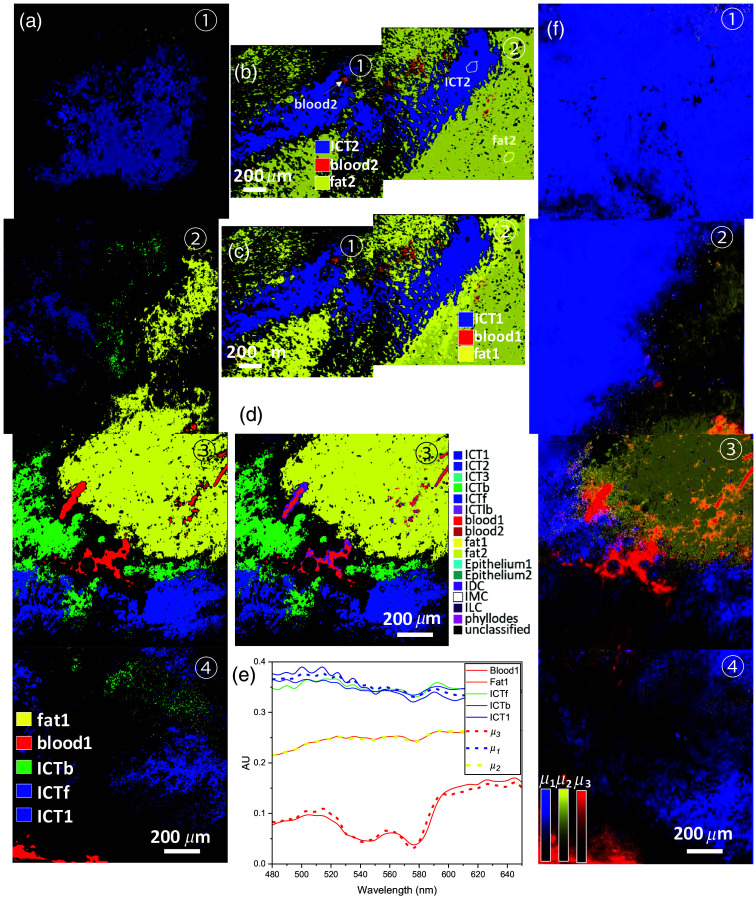
Integrated segmentation maps and spectra of the normal tissue samples. (a) An integrated segmentation map of the normal tissue sample in Fig. 2(a) showing regions classified with fat1, blood1, ICTb, ICTf, and ICT1 using endmembers extracted from a data cube corresponding to FOV3. (b) An integrated segmentation map on a group of contiguous data cubes from the sample Fig. 2(b), showing blood (red), ICT (blue), and fat (yellow) regions classified with their endmembers locally extracted from the same sample. (c) An integrated segmentation map with their endmembers extracted from a data cube of a different sample. (d) A total integrated segmentation map of the FOV3 of the sample Fig. 2(a) computed with all endmembers and with corresponding OSCAs determined from various samples. (e) The top three most abundant endmembers (dotted lines in blue, orange, and red) identified by the unsupervised K-means analysis on the same data cubes whose supervised segmentation map is shown in (a). Endmembers from the supervised algorithm are shown as well for comparison. (f) Unsupervised abundance maps for the first three dominant clusters are shown in different colors.

The fill factor plot for various tissue types in Fig. 6(a) exhibits a sigmoidal pattern with a trend that the fill factor increases in $\overset{¯}{\beta_{i}}$ as more pixels with less similarity between ${\vec{\mu }}_{i}$ and $\overset{\to }{R}(m,n)$ are counted in for each $\overset{¯}{\beta_{i}}$ and eventually converges to 1 for a full coverage fill factor map. To obtain the fill factor plots, discrete $\overset{¯}{\beta_{i}}$ values, 0.05·n(n=0,1,80), were used. The smaller $\overset{¯}{\beta_{i}}$ is, the higher similarity is required for the pixel to be counted in for the fill factor contribution. As ${\overset{\to }{R}}_{l}$ must be orthogonal to ${\vec{\mu }}_{i}$ (i.e., the reference data cube contains no endmember tissue type), $\alpha_{i}^{\mathrm{ort}}$ can be defined with the constraint that satisfies Eq. (4) for all (m,n). As an example, Fig. 7(b) shows a plot of $f_{\text{blood}1}(\overset{¯}{\beta_{i}})$ along with $F_{\text{blood}1}(m,n)$ map at several $\overset{¯}{\beta_{i}}$ values, allowing for finding $\alpha_{\text{blood}1}^{\mathrm{ort}}=0.16$, which is the least $\overset{¯}{\beta_{i}}$ to satisfy $f_{\text{blood}1}({\overset{¯}{\beta }}_{\text{blood}1})\neq 0$ [corresponding image at $\alpha_{\text{blood}1}^{\mathrm{ort}}=0.16$ not shown in Fig. 7(b)]. For all other tissue types, their fill factor plots and $\alpha_{i}^{\mathrm{ort}}$ values are determined likewise and are shown in Fig. 6(a).

Equation (3) with $\alpha_{i}^{\mathrm{th}}$ replaced by $\alpha_{i}^{\mathrm{ort}}$ allows for building a segmentation map of a specific tissue type i. As an example, a segmentation map in Fig. 6(c), $I_{\text{blood}1}^{\mathrm{seg}}(m,n,0.16)$ (i.e., $\alpha_{\text{blood}1}^{\mathrm{th}}=\alpha_{\text{blood}1}^{\mathrm{ort}}=0.16$) superimposed on the corresponding PCI confirms that the algorithm-classified blood map matches to the blood regions in the corresponding PCI with visually identified red color. For a better visibility, the calculated blood segmentation map is overlaid by translating it 50 pixels both in the x- and y-directions. Figure 6(d) shows the calculated coverage ratio versus $\alpha_{\text{blood}1}$, several segmentation maps at various $\alpha_{\text{blood}1}$ are shown as well. Note that the specificity of blood segmentation map becomes worse as $\beta_{i}$ increases far beyond the $\alpha_{i}^{\mathrm{ort}}$ and eventually becomes completely uncorrelated.

Our goal is to establish a library of (${\overset{\to }{\mu }}_{i},\alpha_{i}^{\mathrm{ort}}$) [i.e., (endmember, OSCA) pairs] of multiple tissue types to build an integrated segmentation map showing multiple regions, each of a specific tissue type i. So far, we demonstrated a supervised algorithm to build a segmentation map of only one type, blood, but the technique has been expanded to multiple tissue types. The final integrated segmentation map is a simple superposition of multiple segmentation maps of various tissue types under a constraint that the pixel of interest is classified into a specific tissue type group which gives rise to the least SCA among other SCA values computed for all tissue types so that multicolor segmentation map is

$$\underset{i}{\sum }\{I_{i}^{\mathrm{seg}}(m,n,\alpha_{1,i})⊗C_{i}|\mathrm{Min}(\alpha_{1,i}(m,n))\},$$ (7)

where ⊗ is a color-assigning operator to assign each pixel with a specific color, $C_{i}$ associated with ${\vec{\mu }}_{i}$ which results in the minimum SCA.

Figure 7(a) shows an integrated segmentation map of the normal tissue sample shown in Fig. 2(a). The map was obtained by stitching four segmentation maps individually computed for the four contiguous data cubes with the same set of endmembers locally extracted from one FOV (FOV3). Across the four FOVs, the final segmentation map shows regions classified with fat1, blood1, ICTb, ICTf, and ICT1 assigned with various colors: yellow, green, bright blue, and dark blue, respectively. To construct the maps, we used $\alpha_{i}^{\mathrm{th}}=\alpha_{i}^{\mathrm{ort}}$ for these tissue types. The resulting map demonstrates that: (1) the fat segmentation map (yellow) is continuous across the FOVs 2 and 3; (2) the ICTb is confined near the blood vessels only; (3) ICTf near the fat map; but (4) ICT1 far away from both blood and fat maps, indicative of our supervised technique is sensitive to classify regions with tissue types with endmembers of subtle differences. More importantly, these results verify that the algorithm classifies the target tissue types in other FOVs’ data cubes of the same tissue sample even with locally extracted endmembers.

Next, we demonstrate that our supervised algorithm classifies the target tissue types in other data cubes of different samples. Figure 7(b) shows an integrated segmentation map on a group of contiguous data cubes from the sample Fig. 2(b), showing blood (red), ICT (blue), and fat (yellow) regions classified with their endmembers locally extracted from the same sample. On the other hand, Fig. 7(c) shows an integrated segmentation map with their endmembers, blood1, fat, and ICT1, which were extracted from a data cube of different sample, the FOV3 of the sample in Fig. 2(a). No difference is noticed between two maps in (b) and in (c) regardless whether the endmembers are from the same or different data cube, implying that a library of (${\overset{\to }{\mu }}_{i},\alpha_{i}^{\mathrm{th}}$) determined from one data cube of one sample can be globally applied to all the other samples to build integrated maps classified with the same tissue types if present. To support this claim, Fig. 7(d) shows a total integrated segmentation map of the FOV3 of the sample Fig. 2(a) computed with the entire endmembers and with corresponding OSCAs determined for various endmembers, exhibiting well-classified regions with blood, fat, and ICTs, each assigned with different color. For this map, $\alpha_{i}^{\mathrm{th}}$ (TSCA) for all those tissue types were fixed at $\alpha_{i}^{\mathrm{ort}}$ (OSCA). The assigned color to each tissue type is shown at the right side of the map. This segmentation map shows almost the same color patterns as in Fig. 7(a), verifying the final library of (${\vec{\mu }}_{i},\alpha_{i}^{\mathrm{th}}$) with all locally extracted endmembers effectively classifies various tissue types across different samples. Note that the map should show only tissue types that are present, but IDC-pixels are classified at the perimeter of the blood regions because $\alpha_{1,\mathrm{IDC}}(m,n)<\alpha_{1,\text{blood}1}(m,n)$ in those regions. From histopathology reading, we know that the sample does not contain any IDC, so we observe that $\alpha_{\text{blood}1}^{\mathrm{th}}$ needs to be increased above $\alpha_{\text{blood}1}^{\mathrm{ort}}$ to classify those pixels into the blood type. Further adjustment for an accurate TSCA ($\alpha_{i}^{\mathrm{th}}$) determination would be necessary with a ground truth information on the distribution of the tissue type i.

### 3.3 Unsupervised Approach to Build Segmentation Maps of Various Tissue Types Based on the *K*-Means Algorithm

Building an accurate segmentation map at single-pixel resolution is advantageous for locating tumor margins with microscopic resolution. The supervised approach based on the SAM algorithm classifies pixels into specific tissue types whose endmember is the most similar to the spectrum at that pixel, allowing for segmentation maps with spatially resolved regions classified with various tissue types. In biological tissues, data cubes with pixels with mixed spectra are common, and the spectral unmixing of tumor spectrum from the others is necessary to precisely determine the tumor margin. Toward this goal, an unsupervised classification method based on the K-mean algorithm is demonstrated to build a segmentation map with spatially resolved regions dominantly with carcinoma. Then, by comparing the segmentation maps by the supervised SAM against the unsupervised K-means results, the identity of the unmixed spectra from the K-means algorithm can be validated from the tissue type label by the supervised analysis. Furthermore, the unsupervised algorithm delineates the degree of contribution by each endmember when multiple tissue type signatures are mixed within one pixel.

The K-means algorithm initially involves combining multiple data cubes into one and converting the data cube into a 2D matrix from, $X\in R^{n\times p}$, where n is the number of pixels, and p is the number of wavelengths. Namely, the (i,j)’th entry of X is the reflection intensity of the i’th pixel at the j’th wavelength. Then, the K-means clustering divides n pixels into k clusters and minimizes each cluster’s variances by solving the following problem for a disjoint partition of [1,2,…,n], written as $\left[S=\{S_{1},S_{2},\ldots ,S_{k}\}\right]$:

$$\min\;\overset{k}{\underset{i=1}{\sum }}\underset{j\in S_{i}}{\sum }{\left\|X_{j\cdot }-\mu_{i}^{T}\right\|}_{2}^{2},$$ (8)

where ${\left\|\cdot \right\|}_{2}$ denotes the $L^{2}$ norm (the square root of the sum of the squared vector values), $\mu_{i}^{T}=\frac{1}{\left|S_{i}\right|}\underset{j\in S_{i}}{\sum }X_{j\cdot }$, $\left|S_{i}\right|$ is the cardinality of $S_{i}$, and $X_{j\cdot }$ are row vectors of X. The problem is solved iteratively with initial cluster centers set as k row vectors of X randomly chosen from all distinct row vectors. Aiming at more stable solutions, we ran 10 random starts for each X.^70^ The extracted endmembers are $\left\{\mu_{i};i=1,2,\ldots ,k\right\}$, and the corresponding abundance maps are $\left\{(1_{S_{i}}(j){)}_{j=1}^{n};i=1,2,\ldots ,k\right\}$, where $1_{S_{i}}(j)=1$ if $j\in S_{i}$ and $1_{S_{i}}(j)=0$ otherwise. Abundance maps with more continuous values are obtained by solving

$$m_{j}=\underset{m\in R^{k},m\geq 0}{\arg\;\min}\|Um-X_{j\cdot }^{T}{\|}_{2},\;j=1,\ldots ,n,$$ (9)

where $U=(\mu_{1},\mu_{2},\ldots ,\mu_{k})\in R^{p\times k}$. Let $M=(m_{1},m_{2},\ldots ,m_{n}{)}^{T}\in R^{n\times k}$, then the i’th column of M is the abundance map corresponding to endmember $\mu_{i}$ (i=1,…,k), respectively.

Here, with k=5 and order 5 clusters using the percentage of variance determined by each cluster, the top five most abundant endmembers were determined. Three abundance maps with the top three most abundant endmembers, $\mu_{i}(i=1,2,3)$), each in different color, blue ($\mu_{1}$), yellow ($\mu_{2}$), and red ($\mu_{3}$), are combined and is shown in Fig. 7(f). Each intensity scale bar in color represents the similarity (i.e., closest to the cluster center) of the spectrum at a pixel to the endmember $\mu_{i}$, quantifying the degree of spectral contribution by corresponding endmember. For example, some pixels in the combined map are mixed with red, yellow, blue in different ratios, indicative of subpixel spectral mixing at those pixels, implying spectral unmixing at a single pixel resolution (1.95  μm) is possible by quantifying the ratio among them. As unsupervised approach does not rely on *a priori* information on the tissue type for each endmember, the results need to be validated and each endmember needs to be labeled with a specific tissue type. Comparing the two segmentation maps in Figs. 7(a) and 7(f), the classified pattern with each color is in good agreement with each other, enabling assignment of a specific tissue type to each $\mu_{i}$: blue to ICT, yellow to fat, and red to blood. These labeled endmembers are presented in Fig. 7(e) (dotted lines in blue, orange, and red) along with supervised endmembers for comparison, exhibiting the endmembers from the supervised and unsupervised algorithms are in good agreement to justify the assignment.

### 3.4 Comparison between the Supervised and Unsupervised Algorithms in Establishing a Segmentation Map of Carcinoma

**Fig. 8:**
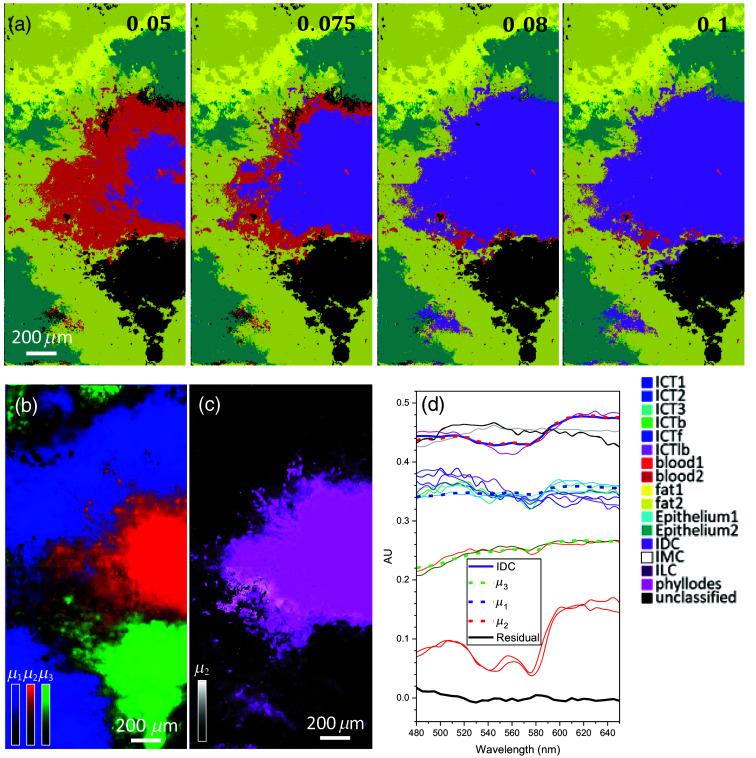
Segmentation maps of the IDC. (a) Integrated supervised segmentation maps on two contiguous data cubes from the sample with IDC [Fig. 2(g)]. The numbers in the upper right corners are various spectral correlation angles, $\alpha_{i}^{\mathrm{th}}$ for the detection of IDC. (b) Three abundance maps with the top three most abundant endmembers, $\mu_{i}$ (i=1, 2, 3), each in different colors, blue ($\mu_{1}$), red ($\mu_{2}$), and green ($\mu_{3}$), are combined. (c) A superimposed image of the unsupervised map in grayscale with the purple supervised IDC segmentation map at $\alpha_{\mathrm{IDC}}^{\mathrm{th}}=\alpha_{\mathrm{IDC}}^{\mathrm{ort}}=0.08$ to confirm their spatial colocalization. (d) Endmembers by different methods. The color for each endmember curve is the same that used for the color for its corresponding

Figure 8(a) shows total integrated supervised segmentation maps on two contiguous data cubes from the sample with IDC [Fig. 2(g)]. Multiple maps shown are at various $\alpha_{\mathrm{IDC}}^{\mathrm{th}}$ (shown with the number at the top of each map) computed by the supervised SAM algorithm while keeping the $\alpha_{i}^{\mathrm{th}}$ values of other tissue types fixed at $\alpha_{i}^{\mathrm{ort}}$. The maps computed with all endmembers and corresponding OSCAs exhibit well-classified regions of IDC (purple) and other tissue types, including blood, epithelium, and fat. The assigned color to each tissue type is shown below the panel at $\alpha_{\mathrm{IDC}}^{\mathrm{th}}=0.1$. The maps display only tissue types that are detected and classified, revealing that other tumor types (ILC, IMC, and phyllodes) are not present, verifying that our supervised algorithm with the library of (${\overset{\to }{\mu }}_{i},\alpha_{i}^{\mathrm{ort}}$) for all tissue types identifies an IDC subtype. Note that more peripheral pixels around the IDC region are classified into blood-segmented group as $\alpha_{\mathrm{IDC}}^{\mathrm{th}}$ decreases below $\alpha_{\mathrm{IDC}}^{\mathrm{ort}}$ (see the maps with $\alpha_{\mathrm{IDC}}^{\mathrm{th}}=0.05$ and 0.075) because $\alpha_{1,\text{blood}1}(m,n)<\alpha_{1,\mathrm{IDC}}(m,n)$ in that region. This phenomenon implies spectral mixing of ${\vec{\mu }}_{\mathrm{IDC}}$ and ${\overset{\to }{\mu }}_{\text{blood}1}$ at those pixels, being consistent with an expectation that high blood concentration is associated with the IDC region due to increased micro vessel density (hot spot) in the vicinity.^71^^,^^72^ Note that the IDC-classified area remains the same at $\alpha_{\mathrm{IDC}}^{\mathrm{th}}=0.1>\alpha_{\mathrm{IDC}}^{\mathrm{ort}}$ as TSCAs of other tissue types are still higher outside the IDC-classified region.

After analysis with the unsupervised K-means algorithm, three abundance maps with the top three most abundant endmembers, $\mu_{i}(i=1,2,3)$, each in different color, blue ($\mu_{1}$), red ($\mu_{2}$), and green ($\mu_{3}$), are combined and shown in Fig. 8(b). Five abundance maps corresponding to the top five dominant endmembers and their coverage percentages are presented in Fig. S1 in the Supplemental Material. Note that the K-means algorithm was applied on a hypercube combined with the two data cubes. Each intensity scale in the color bar represents the similarity (i.e., closest to the cluster center) of the spectrum at a pixel to the endmember $\mu_{i}$, quantifying the degree of spectral contribution by the corresponding endmember. Comparing the two segmentation maps in Fig. 8(a) (at $\alpha_{\mathrm{IDC}}^{\mathrm{th}}=0.08$) and Fig. 8(b), the classified purple regions in (a) and red region in (b) spatially colocalize, suggesting that $\mu_{2}$ is the endmember of the IDC. Figure 8(c) shows the abundance map for $\mu_{2}$ (grayscale) superimposed on the supervised map at $\alpha_{\mathrm{IDC}}^{\mathrm{th}}=0.08$ (see the purple region), confirming their spatial colocalization. In Fig. 8(d), the plot of various endmembers from both supervised and unsupervised results, the IDC endmember plot by unsupervised algorithm (solid purple curve), and the plot of $\mu_{2}$ (dashed red) are in good agreement, confirming that $\mu_{2}$ represents IDC. To quantify the agreement, the residual ratio of the supervised IDC endmember versus the unsupervised for the presented sample, ($\mu_{\mathrm{IDC}}^{\text{Sup}}(\lambda )-\mu_{\mathrm{IDC}}^{\text{Unsup}}(\lambda ))/\mu_{\mathrm{IDC}}^{\text{Unsup}}(\lambda )$, is plotted in the solid black line in the same plot, to confirm <2% difference. However, identifying the counterparts of $\mu_{1}$ and $\mu_{3}$ from endmembers by supervised results is not trivial as the patterns do not colocalize each other, implying that sub-pixel spectral mixing in other tissue types is severe in this tissue sample. In fact, although $\mu_{1}$ (blue dashed) and $\mu_{3}$ (green dashed) resemble the ICT and fat endmember, respectively, their plots in Fig. 8(d) show subtle differences. To identify matching endmember pairs of other tissue types, further spectral unmixing needs to be performed.

**Fig. 9:**
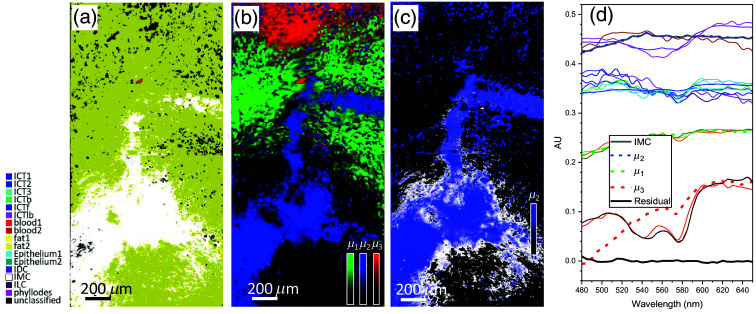
Segmentation maps of the IMC. (a) An integrated supervised segmentation maps on two contiguous data cubes from the sample with IMC [Fig. 2(f)]. (b) Three abundance maps with the top three most abundant endmembers, $\mu_{i}$ (i=1, 2, 3), each in different color, green ($\mu_{1}$), blue ($\mu_{2}$), and red ($\mu_{3}$), are combined. (c) A superimposed image of the unsupervised map for $\mu_{2}$ in blue with the white supervised IDC segmentation map to confirm their spatial colocalization. (d) Endmembers by different methods. The color for each endmember curve is the same that used for the color for its corresponding abundance map in Fig. 8(b).

Further comparative results presented in Fig. 9 demonstrate the classification of another carcinoma subtype, IMC. Figure 9(a) shows a total integrated supervised segmentation map on two contiguous data cubes from the sample with IMC [Fig. 2(f)]. The maps computed with all endmembers and corresponding OSCAs exhibit well-classified regions of IMC (white) and other tissue types in different colors. The color of each tissue type is shown next to Fig. 9(a). The map displays only tissue types that are detected and classified, revealing that other tumor types (ILC, IDC, and phyllodes) are not present, verifying that our supervised algorithm with the library of (${\overset{\to }{\mu }}_{i},\alpha_{i}^{\mathrm{ort}}$) for all tissue types identifies the IMC subtype. After analysis with the unsupervised K-means algorithm, the three abundance maps with the top three most abundant endmembers, $\mu_{i}(i=1,2,3)$, each in a different color, green ($\mu_{1}$), blue ($\mu_{2}$), and red ($\mu_{3}$), are combined in Fig. 9(b). Five abundance maps corresponding to the top five dominant endmembers and their coverage percentages are presented in Fig. S2 in the Supplemental Material. Note that the K-means algorithm was applied on a hyperpercube combined with the two data cubes. Comparing these two maps, the classified white regions in (a) and blue region in (b) colocalize spatially, suggesting that $\mu_{2}$ is the endmember of IMC. Figure 9(c), the abundance map for $\mu_{2}$ (blue) superimposed on the supervised IMC map (see the white region), confirms their spatial colocalization. In Fig. 9(d), the plot of various endmembers from both supervised and unsupervised results, the IMC endmember plot by unsupervised algorithm (solid gray curve) and the plot of $\mu_{2}$ (dashed blue) are in good agreement, confirming that $\mu_{2}$ is for IMC. To quantify the agreement, the residual ratio of the supervised IDC endmember versus unsupervised for the presented sample, ($\mu_{\mathrm{IMC}}^{\text{Sup}}(\lambda )-\mu_{\mathrm{IMC}}^{\text{Unsup}}(\lambda ))/\mu_{\mathrm{IMC}}^{\text{Unsup}}(\lambda )$, is plotted in black solid line in the same plot, to illustrate <2% difference.

### 3.5 Perspective on the Potential of Expert-Validated Unsupervised Algorithm for Image-Guided Surgery with High Accuracy

We have demonstrated that the manually extracted, and pathologist-validated, endmembers of known tissue types including carcinoma subtypes and their associated threshold SCA for classification make a good reference library which is essential to validate endmembers computed by the unsupervised algorithm. The library can be used to label classified endmembers by the unsupervised analysis to identify a target carcinoma endmember and its associated abundance map. This abundance map defines the spatial distribution of the target carcinoma. Image analyses with a supervised algorithm can be automated through a computation pipeline with feedback from the library information. This automated process can be performed in real-time with a predefined library to enable microscopic evaluation of the tumor margin in the operating room, substantially reducing the risk of a second surgery in BCS. This approach with a validated library may be applied to other precision medicine applications, such as image-guided surgeries, which require high accuracy. For instance, selective eradication of tumor cells in micro-neurosurgeries involving glioblastoma and neurothekeoma may be assisted by the real-time HSI analysis technique to improve the prognosis and the quality of life of the patient.

## 4 Conclusion

HSDFM and data cube analysis algorithms demonstrate successful classification of various tissue types and the detection of carcinoma regions in human post-lumpectomy breast tissues excised by breast-conserving surgeries. Two classification algorithms, supervised and unsupervised algorithms, are discussed and employed to identify regions with carcinoma subtypes of IDC and IMC present in the tissues. The two carcinomas’ unique endmembers used by the two methods agree to <2% residual error margin. One of the key motivations for this report is to demonstrate a robust procedure for the validation of an unsupervised algorithm with the essential set of parameters based on the ground truth information. We have demonstrated that a trained library of the histopathology-guided endmembers and associated threshold SCAs computed against well-defined reference data cubes serve such parameters. This library is collected under an environment with no ambient background and may be instrumental in developing or validating more advanced unsupervised data cube analysis algorithms, such as effective neural networks for efficient subtype classification, as has been demonstrated in recent work elsewhere.^73^^,^^74^

## Supplementary Material



## Data Availability

Data used in this article can be made available by a reasonable request to the corresponding author. Materials used in this article can be made available through a materials transfer agreement.
