# Supplementary material for: Hyperspectral dark-field microscopy of human breast lumpectomy samples for tumor margin detection in breast-conserving surgery
Source: J Biomed Opt. 2024 May 7;29(9):093503. doi: 10.1117/1.JBO.29.9.093503 (PMC11075096; doi:10.1117/1.JBO.29.9.093503)
Supplement: Supplementary file 1 [file JBO_029_093503_SD001.pdf]

Supplemental content

Hyperspectral dark-field microscopy of human breast lumpectomy samples for tumor margin detection in breast-conserving surgery

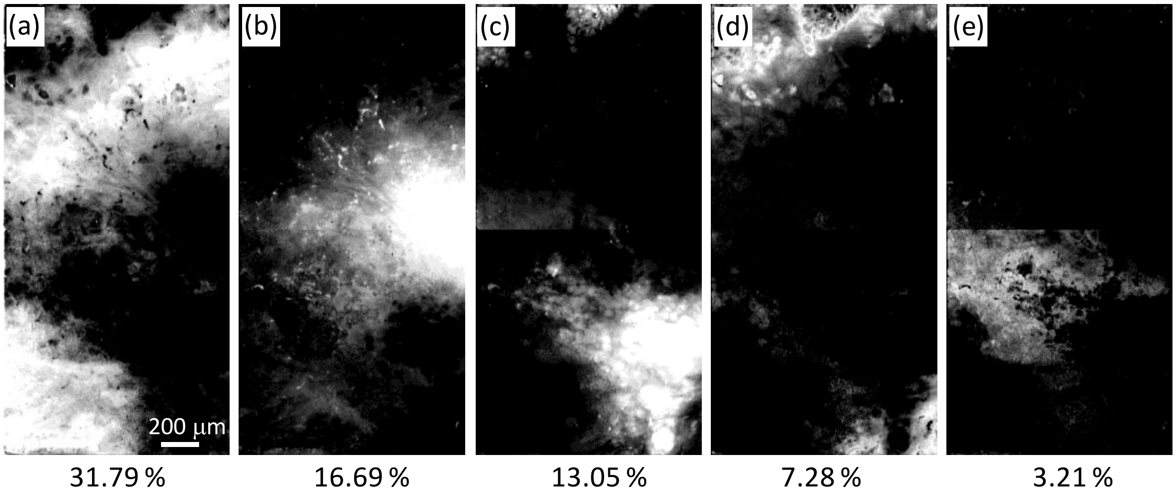

Fig. S1 Abundance maps in percentage of the top 5 endmembers from the K-means unsupervised analysis of the invasive ductal carcinoma data cubes.

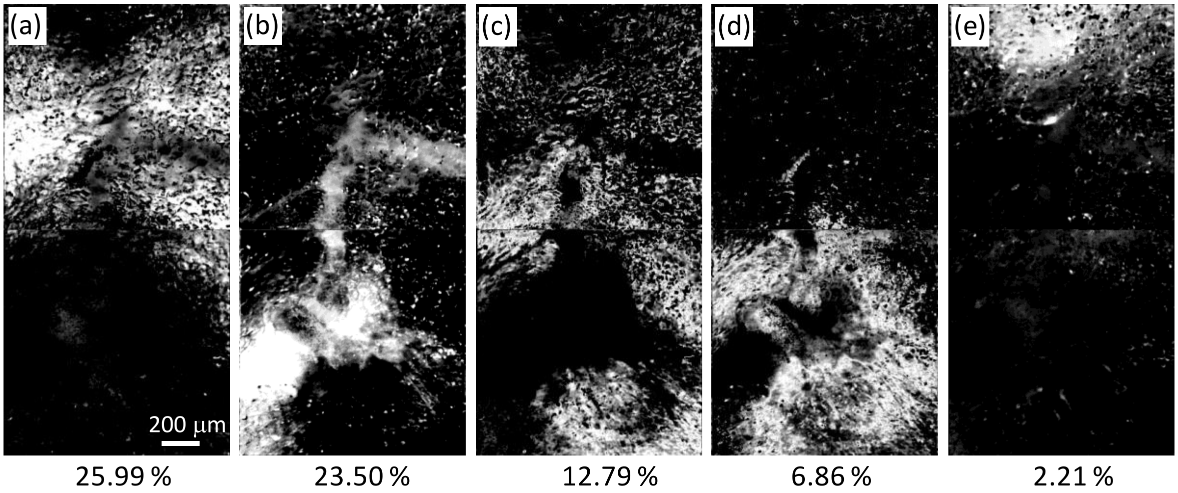

Fig. S2 Abundance maps in percentage of the top 5 endmembers from the K-means unsupervised analysis of the invasive mucinous carcinoma.

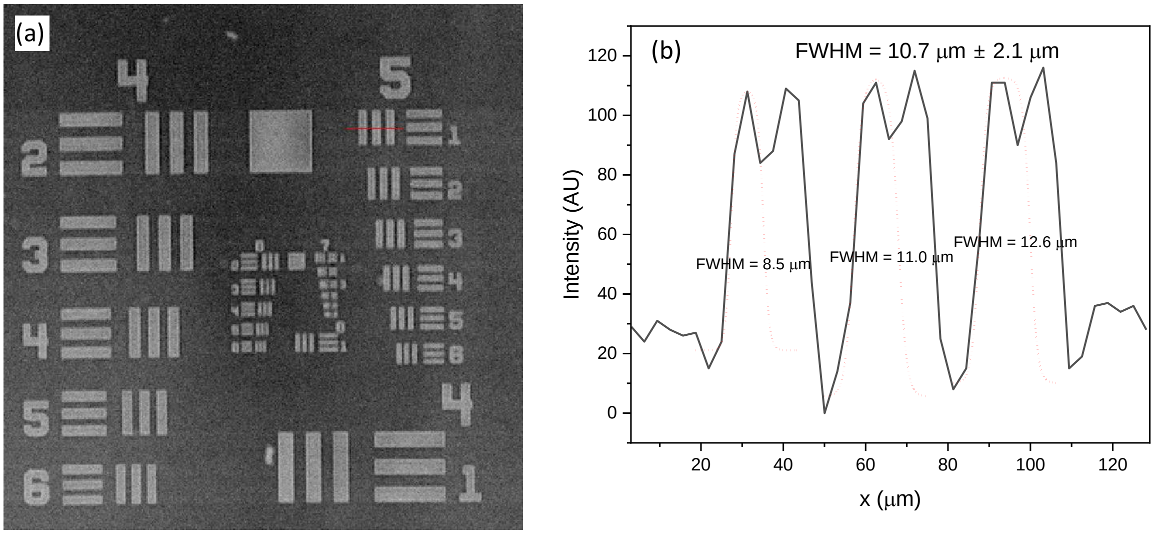

Fig. S3 The spatial resolution of the hyperspectral dark-field microscopy. (a) A dark-field image of a USAF 1951 resolution target with a red line from which an intensity profile across the three vertical patterns of 15.63 μm in width is shown with a black solid line in panel (b). The intensity in this image at each pixel is a summed intensity across all wavelength bands at the pixel. (b) The intensity profile across the left edge of each vertical pattern is fitted with a rising sigmoidal curve to get the full width of the half maximum (FWHM) of the intensity profile of the single peak intensity profile constructed with the rising and its symmetric falling curve. The mean and standard deviation of the three FWHM values were calculated from the three edge profile curves shown as red dotted lines.
